# A one-year follow-up study on dynamic changes of leukocyte subsets and virus-specific antibodies of patients with COVID-19 in Sichuan, China

**DOI:** 10.7150/ijms.71286

**Published:** 2022-06-27

**Authors:** Renjie Xu, Bennan Zhao, Lijuan Lan, Yaling Liu, Yalun Li, Liangshuang Jiang, Shuiping Dai

**Affiliations:** 1 Institute of Respiratory Health, West China Hospital, Sichuan University, Chengdu, Sichuan, China.; 2 Department of Comprehensive Internal Medicine, the Public and Health Clinical Center of Chengdu, Chengdu, Sichuan, China.; 3 Department of Respiratory and Critical Care Medicine, West China Hospital, Sichuan University, Chengdu, Sichuan, China.; 4 Center of Gerontology and Geriatrics, West China Hospital, Sichuan University, Chengdu, Sichuan, China.

**Keywords:** SARS-CoV-2, Virus-specific antibody, Leukocyte subsets, Follow-up, Vaccine

## Abstract

**Background:** SARS-CoV-2 infection causes immune response and produces protective antibodies, and these changes may persist after patients discharged from hospital.

**Methods:** This study conducted a one-year follow-up study on patients with COVID-19 to observe the dynamic changes of circulating leukocyte subsets and virus-specific antibodies.

**Results:** A total of 66 patients with COVID-19 and 213 healthy patients with inactivated SARS-CoV-2 vaccination were included. The virus-specific total antibody, IgG and IgM antibody of patients after one year of recovery were higher than those of healthy vaccinated participants (94.13 vs 4.65, 2.67 vs 0.44, 0.09 vs 0.06, respectively) (*P* < 0.001). Neutrophil count (OR = 1.73, 95% CI: 1.10-2.70, *P* = 0.016) and neutrophil-to-lymphocyte ratio (OR = 1.59, 95% CI: 1.05-2.41, *P* = 0.030) at discharge were the influencing factors for the positivity of virus-specific IgG antibody in patients after one year of recovery. The counts of CD4+ and CD8+ T, B and NK cells increased with the time of recovery, and remained basically stable from 9 to 12 months after discharge. After 12 months, the positivity of IgG antibody was 85.3% and IgM was 11.8%, while the virus-specific antibody changed dynamically in patients within one year after discharge.

**Conclusions:** The SARS-CoV-2 specific antibody of recovered patients showed dynamic fluctuation after discharge, while the leukocyte subsets gradually increased and basically stabilized after 9 months.

## Introduction

Severe acute respiratory syndrome coronavirus 2 viruses (SARS-CoV-2) has caused a huge negative impact not only on global public health, but also on the economic status of nations and individuals 1, 2. As of September 2021, the cumulative number of cases with COVID-19 reported globally is over 224 million and the cumulative number of deaths is over 4.6 million, over 123 thousand cases and over 5 thousand deaths in China (http://2019ncov.chinacdc.cn/2019-nCoV/) 3. SARS-CoV-2 specific antibodies are critical for predicting disease severity and survival and preventing reinfection 4. A rhesus macaque model of SARS-CoV-2 infection suggests that primary SARS-CoV-2 exposure protects against subsequent reinfection 5. Neutralizing antibodies are generated by the humoral immune system and reduce the viral load by binding to spike protein components, which are the proteins secreted by plasma cells and serve as an important part of the defense mechanism 6. The neutralizing antibodies are a standard method to evaluate serum protection against SARS-CoV-2 infection and to explore whether serum is still protective against reinfection 7, 8. However, at present, the virus neutralization test or pseudovirus-based neutralization test needs to be performed in a specialized biosafety level 2 or 3 laboratory and requires the use of live virus, which is not suitable for the follow-up of patients with COVID-19 in general hospitals 9, 10. Previous studies found that SARS-CoV-2 specific antibodies correlated positively with virus neutralizing antibodies 11, 12. Therefore, this study tracked the SARS-CoV-2 specific total antibodies, IgG and IgM antibodies in patients with COVID-19 rehabilitation for 1 year to investigate the persistence of protective humoral responses.

Immunocytes play a fundamental role in viral infections 13. Natural killer (NK) cells exert anti-SARS-CoV-2 activity but are functionally impaired in severe COVID-19 14, and antibodies induced by SARS-CoV-2 infection can trigger significant NK cell-mediated antibody dependent cellular cytotoxicity 15. CD4+ T cells have the ability to instruct B cells, help CD8+ T cells, recruit innate cell, have direct antiviral activities, and facilitate tissue repair; CD8+ T cells are critical for clearance of viral infections with the ability to kill infected cells 16. Multiple studies have reported that the drastically reduced numbers of NK cells, CD4+ and CD8+ T cells in COVID-19 patients was associated with severity of the disease 17-19. SARS-CoV-2 elicits a robust B cell response, and then viral-specific IgM, IgG, IgA and neutralizing IgG antibodies can be detected in the peripheral blood of patients within a few days after infection 13, 20. Previous reports suggested a significant decrease in numbers of NK cells, B cells, CD4+ and CD8+ T cells in patients with severe COVID-19 21, 22, and the absolute number of these immunocytes increased during the convalescent period 23. A six-month follow-up study found that the lymphocyte counts increased in 97% patients with lymphocytopenia 24. Recently, one study reported that SARS-CoV-2 specific T cell immune responses remained stable up to one year after recovery 25. However, the dynamic changes of NK cells and multiple lymphocytes subsets in patients with COVID-19 after one year of recovery have not been clarified.

The virus vaccine can induce immune responses and the production of protective antibodies. Inactivated virus vaccine candidates have been approved for emergency use in China 26. PiCoVacc and BBIBP-CorV have been reported to induce substantial antibody production without T cell responses, which means that they are effective and safe 27, 28. After vaccination, the virus specific antibodies were positively correlated with neutralizing antibodies, and these two kinds of antibodies were positively correlated with CD4+ T cell responses 12, 29. The technical guidelines for SARS-CoV-2 vaccination in China (1st edition) suggested that patients with previous infection with COVID-19 could be vaccinated after 6 months 30. Previous investigations shown that although viral-specific humoral ant T cell responses could last up to 6 to 8 months, they decreased slightly^24^, ^31^. What about the virus specific antibodies and lymphocytes in patients with COVID-19 after one year of recovery and do they need to be vaccinated?

Therefore, this study conducted a one-year follow-up study on COVID-19 recovery patients in Sichuan, China, to observe the dynamic changes of SARS-CoV-2 specific antibodies and leukocyte subsets (NK cells, B cells, CD4+ and CD8+ T cells) with the recovery time, and to analyze the related factors affecting the viral-specific antibodies after one year of recovery. We also compared the viral-specific antibodies of patients after one-year recovery with those of vaccinated healthy people to explore whether patients need to be vaccinated one year after recovery.

## Material and methods

### Populations

This study was a multicenter study, which included local patients with COVID-19 diagnosed in Sichuan Province and healthy people vaccinated with SARS-CoV-2 vaccine. Inclusion criteria for patients with COVID-19: 1) local patients diagnosed in Sichuan Province from January 2020 to March 2020; 2) age ≥ 18 years; 3) the SARS-CoV-2 specific antibody test was completed after 1 year of recovery. Exclusion criteria: 1) refused to join the study or sign informed consent; 2) no SARS-CoV-2 specific antibody data after 1 year of recovery was obtained. In order to compare the SARS-CoV-2 specific antibody levels of patients after 1 years of recovery with healthy people vaccinated with the SARS-CoV-2 vaccine, health care workers, from the Chengdu Public Health Clinical Medical Center, inoculated with inactivated virus vaccine were also included in this study. Inclusion criteria for healthy people: 1) age ≥ 18 years; 2) two dose (of a two-dose schedule) of the inactivated SARS-CoV-2 vaccine have been completed. Exclusion criteria: 1) refused to join the study or sign informed consent; 2) the SARS-CoV-2 specific antibody test was refused. A total of 66 patients diagnosed with COVID-19 in Sichuan Province from January 2020 to March 2020 and 213 healthy people in Chengdu Public Health Clinical Medical Center were included in this study. Patients with SARS-CoV-2 infection were admitted to 14 hospitals in Sichuan Province. After discharge, they were followed up for 1 year in West China Hospital or Chengdu Public Health Clinical Medical Center. Some patients completed follow-up examination in Chengdu Public Health Clinical Medical Center at half a month, 3 months, 6 months and 9 months after discharge. All patients diagnosed with COVID-19 were confirmed with a positive RT-PCR SARS-CoV-2 test and the criteria for discharge and disease severity status were defined by Diagnosis and Treatment Program of New Coronary Pneumonia. 7^th^ ed National Health Commission of the People's Republic of China 32. This study was registered at the China clinical trial registration center (ChiCTR2000034563), approved by the ethics committee of Chengdu Public Health Clinical Medical Center (pj-k2020-06-01), and all subjects have signed informed consent.

### Materials

For patients with COVID-19, the following data were collected: 1) general clinical data, such as gender, age, disease severity status, comorbidities, symptoms, interval days from onset to admission (onset days), the interval between the onset of the disease and the first negative viral nucleic acid test (negative days), hospital days and therapy methods; 2) blood routine data (neutrophil, lymphocyte, platelet count), C-reactive protein (CRP), leukocyte subsets data (CD4 + T cells, CD8 + T cells, B cells, NK cells) at admission and discharge; 3) the SARS-CoV-2 specific antibodies and leukocyte subsets at each time point. The following data were collected for healthy people vaccinated with SARS-CoV-2 vaccine: 1) basic data, such as gender, age, interval months from completion of vaccination to antibody examination; 2) leukocytes, lymphocytes, SARS-CoV-2 specific antibodies.

Two-step capture immunoassay chemiluminescence kits (Innodx Biotech, Xiamen, China) were used to detect IgM and IgG antibodies produced against the RBD protein of SARS-CoV-2 virus spike protein in serum or plasma, with i3000 automatic chemiluminescence immunoassay (Maccura biotechnology, China). Total antibodies referred to the antibodies produced against the RBD protein of SARS-CoV-2 virus spike protein in serum or plasma, including IgG, IgM and IgA antibodies, which was detected by caris200 automatic chemiluminescence immunoassay (Wantai biopharm, China). All tests were conducted according to the instructions and under strict biosafety conditions. The antibody titer was tested once per serum sample. Antibody titers were presented as the measured chemiluminescence values divided by the cut-off (cut-off index, COI), which value was defined by the instructions. COI <1 was regarded as negative, and COI >1 was regarded as positive. The circulating leukocyte subset counting was performed with DxFLEX flow cytometry (BECKMAN COUNLTER Life Science, America). To determine the leukocyte subsets, heparin-anticoagulated whole blood samples were collected and stained with 1) CD45RO-BV421 (BioLegend, San Diego, CA); 2) CD3-PerCP (BD Biosciences, San Jose, CA); 3) CD4-APC-Cy7 (eBioscience, San Diego, CA); 4) CD8-APC (BD Biosciences, San Jose, CA); 5) CD19-PE (BD Biosciences, San Jose, CA); 6) CD56-FITC (BD Biosciences, San Jose, CA). Leukocyte was defined by CD45+, T cell by CD3+, B cell by CD19+, NK cell by CD56+. All participants have completed two dose (of a two-dose schedule) of the inactivated SARS-CoV-2 vaccine (Sinovac, with National Institute for Communicable Disease Control and Prevention, China or Beijing Institute of Biological Products, Sinopharm, with Institute of Viral Disease Control and Prevention, China). All operations were carried out in accordance with the instructions.

### Statistical Analysis

Kolmogorov-Smirnov test was used to analyze the distribution of continuous variables. Normal distribution variables were compared by t-test and represented by mean (standard deviation), non-normal distribution variables by Mann-Whitely test and represented by median (inter-quartile range, IQR). Categorical variables were compared using chi square test. In the correlation analysis, Logistic regression test was used when the dependent variable was classified variable; Spearman rank correlation was used for two non-normal distribution variables. Repeated measures analysis of variance (ANOVA) was used for data analysis at different follow-up time points. *P* value < 0.05 was considered to define statistical significance. All analyses were performed by SPSS 22.0 software (Chicago, IL, USA) and R Studio (version 4.1.0).

## Results

### The viral-specific antibody of patients with COVID-19 after one-year recovery was higher than that of healthy people after vaccination

A total of 66 patients aged from 19 to 76 years (47.09 ± 13.61) were included in this study, including 33 males (50%). As shown in Table S1, among these patients included, 27 (47.37%) had comorbidities, and the most common comorbidity was hypertension (11, 19.30%), followed by chronic liver disease (11, 19.30%); 51 (94.44%) patients had symptoms, of which the most common was fever (37, 68.52%), followed by cough (20,37.04%); 47 (83.93%) patients received antiviral therapy, 49 (87.50%) received interferon therapy, 35 (62.50%) used traditional Chinese medicine and 9 (16.07%) used hormone therapy.

**Figure 1:**
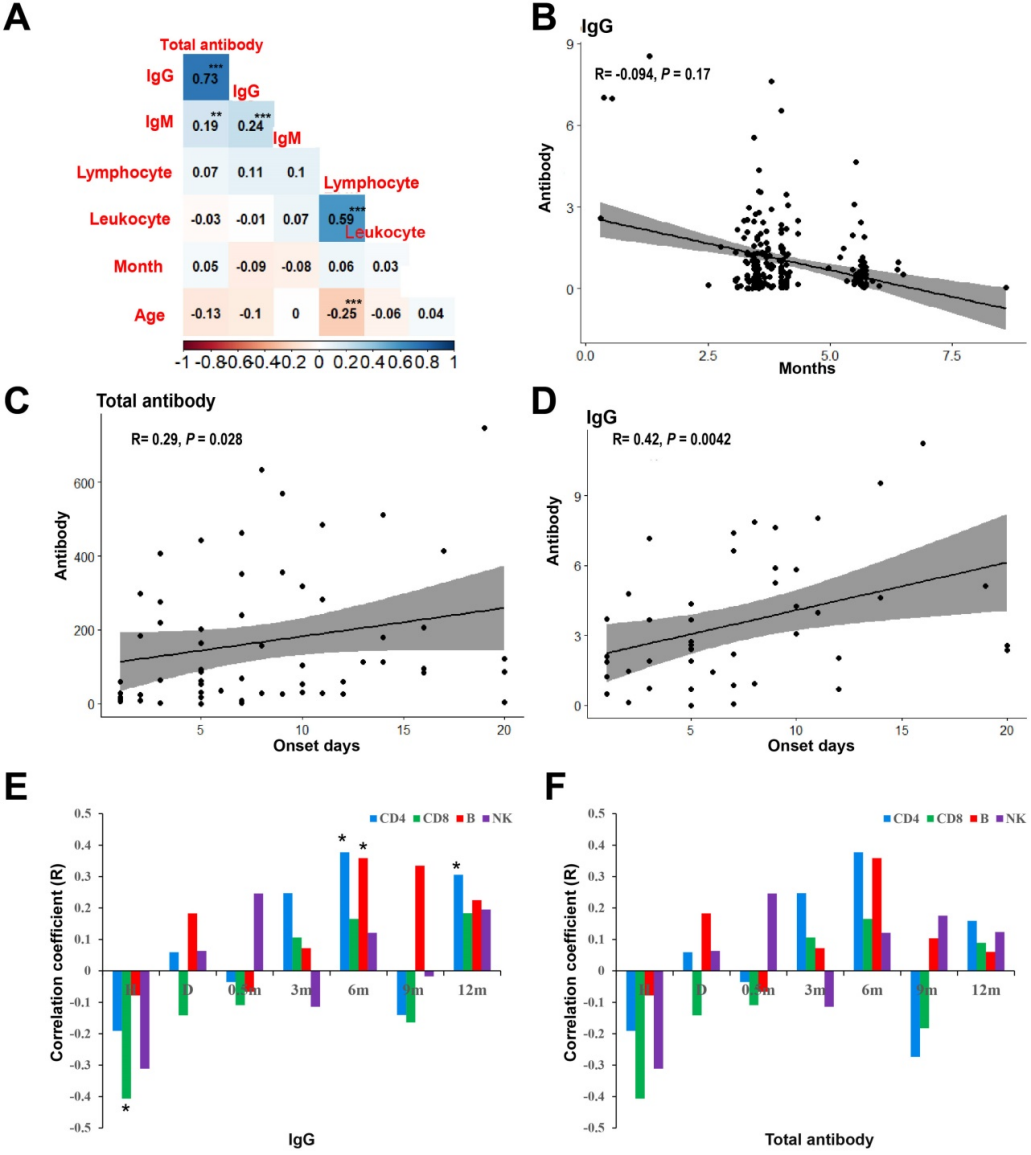
** Antibody level and the correlative factors. (A)** Spearman rank correlation of clinical factors and the levels of SARS-CoV-2 specific antibody of vacinnated healthcare. **(B)** The correlation of viral-specific IgG antibody and the interval months from completion of vaccination to antibody examination. **(C)** Spearman rank correlation of clinical factors and the levels of SARS-CoV-2 specific antibody after 12-months discharge. **(D)** The correlation of viral-specific IgG after 12-months discharge and the interval days from onset to admission. **(E)** The correlation between the counts of lymphoid cell subsets and viral-specific IgG antibody after 12-months discharge. **(F)** The correlation between the counts of lymphoid cell subsets and viral-specific total antibody after 12-months discharge. *** P < 0.001; ** P < 0.01; * P < 0.05; H: at admission; D: at discharge.

In order to compare the SARS-CoV-2 specific antibody of patients with COVID-19 who recovered for one year and healthy people vaccinated with the inactivated virus vaccine, 213 health medical workers who were vaccinated with inactivated virus vaccine in Chengdu Public Health Clinical Medical Center were included in this study, aged from 20 to 58 years, with an mean age of 40.30 year (SD = 8.37), including 24 males (11.30%). Healthy people completed the detection of SARS-CoV-2 specific antibody test after vaccination, and the interval months was from 0 to 8. After two doses of vaccination, healthy people were tested for SARS-CoV-2 specific antibody. The interval between vaccination and detection was 0 ~ 8 months. As shown in Figure 1A, there was a significant positive correlation between viral-specific total antibody and IgG antibody in healthy people after vaccination (R = 0.73, *P* < 0.001). Although there was no significant correlation between interval months and age and viral-specific antibodies, the level of viral-specific IgG antibody in healthy people after vaccination decreased with the interval months (R = -0.09, *P* = 0.17) (Figure 1B).

As shown in Table 1, the SARS-CoV-2 specific antibodies of patients with COVID-19 after one-year recovery were lower than that of 213 healthy people after vaccination. After 1:1 Propensity Score Matching, the viral-specific total antibody, IgG and IgM antibody of patients with COVID-19 were also higher than those of healthy vaccinated people (94.13 vs 4.65, 2.67 vs 0.44, 0.09 vs 0.06, respectively) (*P* < 0.001). The positivity of IgG antibody and IgM in patients was also higher than that of in healthy people with vaccination (53.2 vs 24.2, 6.5 vs 1.5, respectively) (*P* < 0.001).

### Factors associated with SARS-CoV-2 specific antibody in patients with COVID-19 after one year of recovery

Spearman rank correlation was used to analyze the correlation between the baseline characteristics of patients at admission and SARS-CoV-2 specific antibody levels of patients after one year of recovery. As shown in Figure 1C, the interval days from onset to admission was positively correlated with the level of viral-specific total antibody (R = 0.26, *P* = 0.028) and IgG antibody (R = 0.42, *P* = 0.004) (Figure 1D) at 1 year after discharge. The viral-specific total antibody was positively correlated with IgG antibody (R = 0.83, *P* < 0.001), which was consistent to that of that of healthy people after vaccination.

Univariate logistic regression analysis was used to explore the effect of clinical factors in hospital on SARS-CoV-2 specific antibody in patients who recovered for one year. As shown in Table S2, neutrophil count (OR = 1.73, 95% CI: 1.10-2.70, *P* = 0.016) and neutrophil-to-lymphocyte ratio (NLR) (OR = 1.59, 95% CI: 1.05-2.41, *P* = 0.030) were the influencing factors of the positivity of viral-specific IgG antibody in patients after 1 year of recovery, while age, disease severity status and comorbidities had no significant effect on that (*P* > 0.05).

### Kinetics of serum leukocyte subsets and SARS-CoV-2 specific antibody in patients with COVID-19

**Figure 2:**
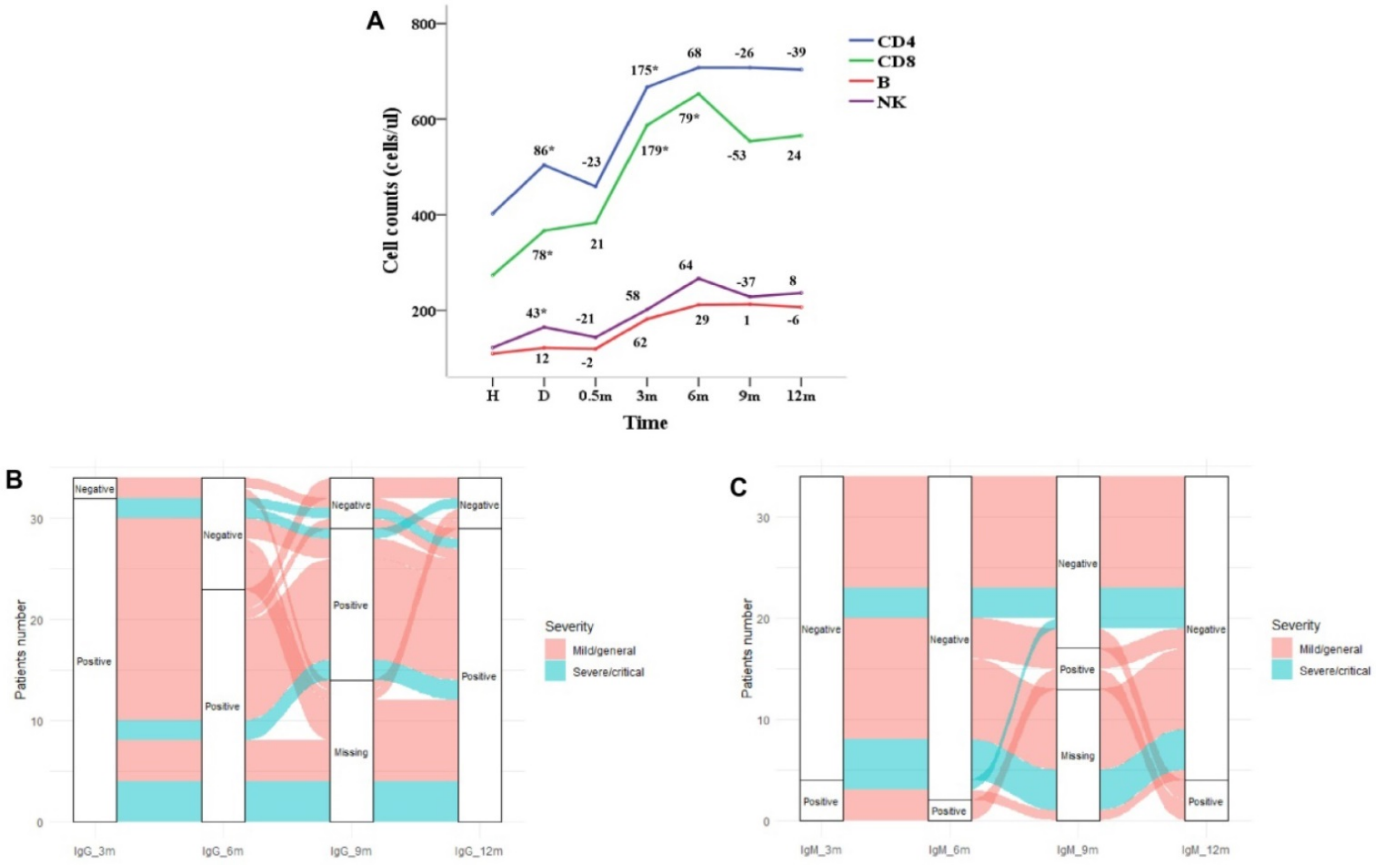
** Dynamic changes of SARS-CoV-2 specific antibody and lymphoid cell subsets with recovery time. (A)** Dynamic changes of lymphoid cell subsets; **(B)** dynamic changes of positivity of viral-specific IgG antibody; (D) dynamic changes of positivity of viral-specific IgM antibody.

Repeated measures ANOVA was used to analysis the leukocyte subsets at different follow-up time points. The dynamics of CD3+ T, CD4+ T, B cells and NK cells over time were shown in Figure 2A. The leukocyte subsets of patients were at the lowest level at admission, and with the treatment of the disease, the counts increased at discharge. The counts of leukocyte subsets reached the highest level at 6 months after discharge and followed by decreased slightly which was basically stable from 9 to 12 months. As shown in Figure 1E, the CD8+ T lymphocytes at admission were negatively correlated with the viral-specific IgG antibody at 1 year after discharge (R = -0.41, *P* = 0.011), and the CD4+ T lymphocytes and B lymphocytes at 6 months were positively correlated with the viral-specific IgG antibody at 1 year after discharge (R = 0.38, *P* = 0.028, R = 0.36, *P* = 0.037, respectively). There was no significant correlation between the counts of leukocyte subsets and viral-specific total antibodies (*P* > 0.05) (Figure 1F).

In this study, 34 patients completed the detection of SARS-CoV-2 specific antibody IgG and IgM at 3, 6 and 12 months after discharge. At 12 months, 29 cases (85.3%) were IgG positive and 4 cases (11.8%) were IgM positive. As shown in Figure 2B and Table S3, IgG antibody of 2 patients with mild/general disease were negative at 3 months, of which 1 patient was negative at 6 and 9 months and turned to positive at 12 months. Among the 32 patients with IgG antibody positive at 3 months, 9 cases turned to negative at 6 months, of which 3 cases turned to positive again at 9 months, 2 cases remained positive at 12 months and 1 case turned to negative. As shown in Figure 2C and Table S3, 30 of the 34 patients were negative for IgM antibody at 3 months, of which 2 cases remained negative at 6 and 9 months, turned to positive at 12 months, and 2 cases were positive at 9 months and turned to negative at 12 months. IgM was positive in 4 patients at 3 months, of which 2 patients were continuously positive at 6, 9 and 12 months.

## Discussion

In this study, the healthy people were vaccinated with inactivated virus vaccine, which safety and efficacy has been proved by phase 1/2 trials 33, 34. In preclinical studies, mice or primates immunized with inactivated virus vaccine elicited virus specific antibodies and neutralizing antibodies 27, 28. In phase 1/2 clinical trials, 14 days after the completion of two doses of vaccination, the conversion rate of serum neutralizing antibody was over 90% 34. An important factor in evaluating the protective power of SARS-CoV-2 vaccine is the titer of neutralizing antibodies in the serum of the vaccinated person. Previous studies found that SARS-CoV-2 specific antibodies correlated positively with virus neutralizing bodies 11, 12. Therefore, the results of the SARS-CoV-2 specific antibodies detection may be of some significance for evaluating the protective power. This study showed that the SARS-CoV-2 specific total antibody and IgG antibody of patients with COVID-19 one year after discharge were significantly higher than those of healthy people vaccinated (*P* < 0.001), suggesting that patients can obtain lasting protection after infection, so the vaccination may not be considered when patients recover from infection for one year.

This study found that the interval days from onset to admission was positively correlated with the SARS-CoV-2 specific total antibody and IgG antibody at 1 year after recovery. It might be due to that over 80% patients in this study received interferon and antiviral therapy after admission, which affected their antiviral immunity. Previous study found that virus specific CD4+ and CD8+ T cells played roles in protective immunity 35. CD4+ T cell are critical for generation of high affinity antibody response, while CD8+ T cell are vital for killing infected cells and mediating viral clearance 36. Neutralizing antibody titers were positively correlated with COVID-19 disease severity in large cohort studies 37, which indicated that higher antigen load drives higher antibody titers 16. We found the CD8+ T lymphocytes at admission were negatively correlated with the SARS-CoV-2 specific IgG antibody at 1 year of rehabilitation, which might be related to the fact that CD8+ T cells reduced the viral burden by killing infected cells 13. Previous studies have shown that as long as there was a strong T cell response, COVID-19 could be controlled without the substantial contribution of neutralizing antibodies 16. In our study, neutrophil count and NLR at discharge were the influencing factors of IgG antibody positive at 1 year of rehabilitation, which was consistent with an observational study 38. Their results indicated that lower IgG levels were associated with a lower lymphocyte percentage and higher neutrophil percentage. The induction of SARS-CoV-2-specific IgA responses linked to neutrophil activation 39. Previous studies also suggested that neutrophil count and NLR were biomarkers associated with COVID-19 disease progression 40. Higher NLR and leukocyte counts might be related to severe cases in some reports 21, 41, while one study found that NLR > 6.11 was associated with lower mortality in patients on corticosteroids 42. The high NLR was associated with excessive levels of reactive oxygen species, which could drive pathological host responses 43. These indicate that we need to pay attention to these factors affecting COVID-19 progression and prognosis, and the specific internal influence mechanism of which needs more research to clarify.

In this study, the counts of CD4+ T, CD8+ T, B and NK cells in patients with COVID-19 increased during the convalescent period, which was consistent with previous study 23. These immune cell counts increased continuously within 6 months after discharge, and remained stable after 9 to 12 months. A study of convalescent patients found that even 2 months after recovery, patients had reduced levels of CD4+ T and B cells 44, which indicated that the recovery of immune cells took longer. We found these immune cell counts continued to increase within 6 months after discharge and remained stable after 9 to 12 months, indicating that the recovery of immune function of patients with COVID-19 might take almost 6 months. However, our results only reflected the overall changes of CD4+ and CD8+ T cells. The SARS-CoV-2 specific CD4+ and CD8+ T cells were more related to protective immunity and immune memory against re-infection 16. A recent study found that convalescent patients presented robust SARS-CoV-2 specific T cell response after seven-month infection 45. Longer follow-up studies are needed to further understand mechanisms of protective adaptive immune responses to COVID-19.

This study found that 85.3% of patients were still positive for SARS-CoV-2 specific IgG antibody at 1 year after discharge. Previous studies found that SARS-CoV-2 specific antibodies were positively correlated with virus neutralizing antibodies 11, 12, which was a standard method to evaluate serum protection against SARS-CoV-2 infection and to explore whether serum still had protective effect on reinfection 7, 8. Therefore, our study suggested that protective humoral responses to SARS-CoV-2 persist up to one year after recovery, which was consistent to previous study 25. At the same time, we observed that the positivity of IgG antibody might change with the time of recovery. For example, two patients were negative for IgG antibody at 3 months after discharge, and one patient was negative at 6 and 9 months and turned positive at 12 months. A previous study found that the seropositivity of IgG was 90.9% in the third month and increased to 95.5% in the sixth month after symptom onset 46, which proved the dynamic change of IgG antibody. However, more basic researches are needed to clarify the reasons for these dynamic changes.

The number of patients with COVID-19 included in this study is small, and larger sample studies are needed to confirm the observations of this study. In this study, the patients were followed up for only one year, and at the end point of follow-up, the SARS-CoV-2 specific antibody of patients was still higher than that of healthy people vaccinated. Therefore, a longer follow-up study is needed to determine the duration of protective antibody of infected patients and the time point when it may be necessary to vaccinate.

## Conclusions

Through a one-year follow-up study on the local patients with COVID-19 in Sichuan Province, we found that the peripheral serum leukocyte counts increased continuously with the extension of recovery time after infection with SARS-CoV-2, and remained stable after 9 to 12 months of recovery. The interval days from onset to admission, the neutrophil count and NLR at discharge were related to SARS-CoV-2 specific antibody titers after one year of recovery. The virus-specific antibody of patients showed dynamic changes in the process of recovery, and the antibody level of patients after one year of recovery was higher than that of healthy people after vaccination.

## Supplementary Material

Supplementary tables.

## Figures and Tables

**Table 1:** Comparison of SARS-CoV-2 specific antibody between COVID-19 patients recovered for 1 year and healthy people after vaccination

	Patients	All healthy people		After match	
		Population	*P*	Population	*P*
Number	66	213		66	
Sex = Male (%)	33 (50.00)	24 (11.30)	<0.001	23 (34.80)	0.111
Age (mean (SD))	47.09 (13.61)	33.84 (7.57)	<0.001	40.30 (8.37)	0.001
qAntibody (median (IQR))	94.13 (249.34)	8.11 (11.95)	<0.001	4.65 (8.58)	<0.001
**IgM (%)**			<0.001		<0.001
Negative	37 (59.70)	209 (98.10)		64 (97.00)	
Positive	4 (6.50)	3 (1.40)		1 (1.50)	
qIgM (median (IQR))	0.09 (0.24)	0.06 (0.06)	<0.001	0.06 (0.06)	<0.001
**IgG (%)**			<0.001		<0.001
Negative	8 (12.90)	140 (65.70)		49 (74.20)	
Positive	33 (53.20)	72 (33.80)		16 (24.20)	
qIgG (median (IQR))	2.67 (3.94)	0.60 (1.07)	<0.001	0.44 (0.84)	<0.001

IQR: interquartile range.

## Abbreviations

SARS-CoV-2: Severe acute respiratory syndrome coronavirus 2 viruses
OR: Odds Ratio
CI: Confidence Interval
IgG: Immunoglobulin G
IgM: Immunoglobulin M
NK: Natural killer
IgA: Immunoglobulin A
RT-PCR: Reverse Transcription-Polymerase Chain Reaction
IQR: inter-quartile range
ANOVA: analysis of variance
NLR: neutrophil-to-lymphocyte ratio
