# Supplementary material for: A one-year follow-up study on dynamic changes of leukocyte subsets and virus-specific antibodies of patients with COVID-19 in Sichuan, China
Source: Int J Med Sci. 2022 Jun 27;19(7):1122–30. doi: 10.7150/ijms.71286 (PMC9339420; doi:10.7150/ijms.71286)
Supplement: Supplementary file 1 — Supplementary tables. [file ijmsv19p1122s1.pdf]

Table S1 Demographics and clinical characteristics of patients with COVID-19 (n=66)

Characteristics	
Age, mean (SD)	47.09 (13.61)
Sex	
Men	33 (50)
Women	33 (50)
Disease severity status	(/57)
Mild/general	47 (82.46)
Severe/critical	19 (33.33)
Comorbidities	27 (/57)
Hypertension	11 (19.30)
Diabetes	9 (15.79)
Cardiovascular diseases	2 (3.51)
Chronic obstructive pulmonary disorder	2 (3.51)
Chronic liver diseases	11 (19.30)
Others	5 (8.77)
Signs and symptoms	51 (/54)
Fatigue	17 (31.48)
Fever	37 (68.52)
Cough	20 (37.04)
Sputum	16 (29.63)
Dyspnea	4 (7.41)
Therapy	(/56)
Hormone	9 (16.07)
Antiviral drugs	47 (83.93)
Interferon	49 (87.50)
Chinese herbs	35 (62.50)

SD: standard deviation. Number (percentage). /number: the number of patients who reported this part of the data.

Table S2 Univariate logistic regression analysis for the influencing factors of SARS-CoV2 viral-specific IgG antibody in patients with COVID-19 after one year of recovery

Factors	OR	95%CI	*P*
Sex	0.64	0.15-2.78	0.552
Age	0.98	0.93-1.04	0.512
Onset days	0.90	0.75-1.07	0.240
Virus negative days	1.01	0.91-1.11	0.913
Hospital days	1.02	0.95-1.10	0.543
Status	0.39	0.04-3.55	0.402
Comorbidities	0.43	0.08-2.37	0.332
Antiviral therapy	1.71	0.18-16.40	0.640
Chinese herbs	1.24	0.26-5.83	0.787
Lymphocyte_H	1.21	0.25-5.94	0.811
Neutrophil_H	1.21	0.92-1.58	0.170
NLR_H	1.07	0.95-1.20	0.258
Hemoglobin_H	0.99	0.94-1.04	0.665
Platelet_H	1.01	1.00-1.02	0.193
PLR_H	1.00	1.00-1.01	0.356
CRP_H	1.01	0.97-1.04	0.680
CD3_H	1.00	0.99-1.00	0.549
CD4_H	1.00	0.99-1.00	0.905
CD8_H	1.00	1.00-1.01	0.139
CD4CD8_H	0.39	0.10-1.56	0.183
B_H	1.01	0.99-1.02	0.377
NK_H	1.00	0.99-1.01	0.873
Lymphocyte_D	0.68	0.11-4.28	0.685
Neutrophil_D	1.73	1.10-2.70	0.016*
NLR_D	1.59	1.05-2.41	0.030*
PLR_D	1.00	0.99-1.01	0.734
Hemoglobin_D	0.98	0.93-1.03	0.462
PLT_D	0.99	0.98-1.01	0.415
CRP_D	1.04	1.00-1.07	0.060
CD3_D	1.00	0.99-1.00	0.518
CD4_D	1.00	0.99-1.00	0.610
CD8_D	1.00	1.00-1.01	0.369
CD4CD8_D	0.180	0.02-1.37	0.099
B_D	0.99	0.97-1.01	0.386
NK_D	1.00	0.99-1.01	0.596

CI: confidence interval; _H: results at admission; _D: results at discharge; NLR: neutrophil to lymphocyte ratio; PLR: platelet to lymphocyte ratio: CRP: C-reactive protein; *: $P < 0.05$.

Table S3 Kinetics of SARS-CoV-2 viral-specific antibody IgG and IgM

Antibody	Status	Follow-up months				Number
		3m	6m	9m	12m	
IgG	Mild/general	Negative	Negative	Missing	Negative	1
	Mild/general	Negative	Negative	Negative	Positive	1
	Mild/general	Positive	Negative	Missing	Negative	1
	Mild/general	Positive	Negative	Missing	Positive	4
	Mild/general	Positive	Negative	Positive	Positive	2
	Mild/general	Positive	Positive	Missing	Positive	4
	Mild/general	Positive	Positive	Negative	Negative	2
	Mild/general	Positive	Positive	Negative	Positive	1
	Mild/general	Positive	Positive	Positive	Positive	10
	Severe/critical	Positive	Negative	Negative	Positive	1
	Severe/critical	Positive	Negative	Positive	Negative	1
	Severe/critical	Positive	Positive	Missing	Positive	4
	Severe/critical	Positive	Positive	Positive	Positive	2
IgM	Mild/general	Negative	Negative	Missing	Negative	8
	Mild/general	Negative	Negative	Negative	Negative	11
	Mild/general	Negative	Negative	Negative	Positive	2
	Mild/general	Negative	Negative	Positive	Negative	2
	Mild/general	Positive	Negative	Missing	Negative	1
	Mild/general	Positive	Positive	Positive	Positive	2
	Severe/critical	Negative	Negative	Missing	Negative	4
	Severe/critical	Negative	Negative	Negative	Negative	3
	Severe/critical	Positive	Negative	Negative	Negative	1
